# Scalp Necrosis as a Late Sign of Giant-Cell Arteritis

**DOI:** 10.1155/2013/231565

**Published:** 2013-01-09

**Authors:** Mohammad Alimohammadi, Ann Knight

**Affiliations:** ^1^Department of Medical Sciences, Uppsala University, Sweden; ^2^Unit of Dermatology, Rheumatology, and Hematology, Uppsala University Hospital, 75185 Uppsala, Sweden

## Abstract

Retinal infarction and scalp necrosis are described as unusual but devastating complications of giant-cell arteritis. We report a patient with this rare complication and emphasize the importance of timely diagnosis and treatment of giant-cell arteritis.

A previously healthy 70-year-old smoking female presented with 10-day history of loss of vision and scalp-tenderness. The event was diagnosed as amaurosis fugax. Fundoscopy indicated a pale oedematous papilla and a temporal peripapillary lozenge-shaped whitish patch on the retina, indicating optic nerve and cilioretinal artery infarction (Figure 1(a)). Her ESR was 69 mm. Giant-cell arteritis (GCA) was suspected and treatment with prednisolone 60 mg daily was initiated. However, a biopsy from the left temporal artery could not confirm the diagnosis and due to a left carotid murmur an ultrasound was performed which revealed a significant carotid stenosis.

An endarterectomy was performed and prednisolone was withdrawn postoperatively as the carotid stenosis was assessed as the cause of patient's symptoms. Within the following two months the patient's vision deteriorated and she developed a bilateral scalp necrosis (Figure 1(b)). A second temporal biopsy confirmed the diagnosis of GCA and prednisolone treatment was restarted. 

GCA is a chronic granulomatous vasculitis of unknown etiology. The prevalence of the disease increase with age. The diagnosis of CGA can be based on clinical findings such as headache, scalp tenderness, jaw claudication, temporal artery abnormalities, elevated erythrocyte sedimentation rate, and biopsy of temporal artery showing granulomatous process [1, 2]. 

Retinal infarction and scalp necrosis are described as unusual but devastating complications to inadequately treated GCA. The present case illustrates that the vasculitis in GCA can affect several extracranial arteries resulting in cilioretinal artery and disc infarction as well as scalp necrosis (Figure 1(c)) [3–5]. The case also highlights importance of timely diagnosis and treatment of GCA. 

Cilioretinal artery and disc infarction can occur in the context of carotid disease [4, 5]. Both giant-cell arteritis and carotid disease can coexist in the same patient and yield some clinical confusion. Even though scalp necrosis due to giant-cell arteritis is rare with around a hundred cases reported, a high level of suspicion must be held for this cutaneous sign in order to initiate prompt therapy to avoid blindness.

## Figures and Tables

**Figure 1:**
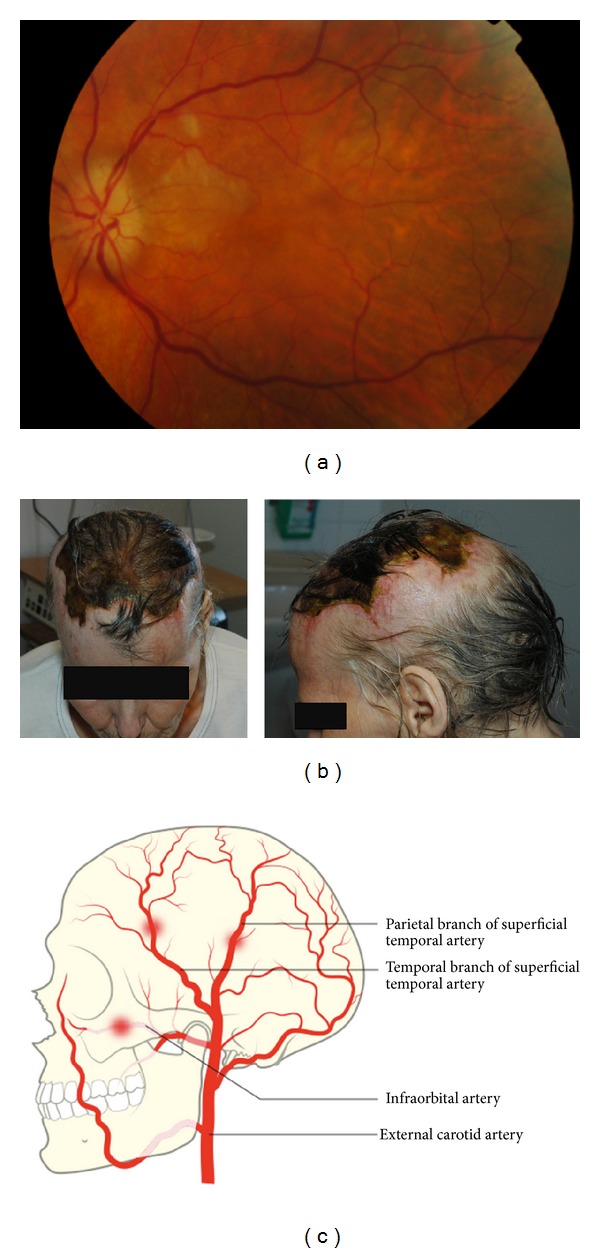
CGA-associated retinal infarction and scalp necrosis—illustration of the underlying pathomechanism. Fundoscopy indicated a pale oedematous papilla and a patch on the retina, indicating optic nerve and retinal infarction (a). Clinical photographs of the patient upon withdrawal of prednisolone (b). Illustration of the branches of external carotid artery supplying retina and the scalp (c).
